# PROTAC Delivery Strategies for Overcoming Physicochemical Properties and Physiological Barriers in Targeted Protein Degradation

**DOI:** 10.3390/pharmaceutics17040501

**Published:** 2025-04-09

**Authors:** Endry Wahyu Syahputra, Hyunji Lee, Hyukjun Cho, Hyun Jin Park, Kwang-Su Park, Duhyeong Hwang

**Affiliations:** 1 College of Pharmacy, Keimyung University, Daegu 42601, Republic of Korea; endrywahyusyahputra@stu.kmu.ac.kr (E.W.S.); hjcho89@kmu.ac.kr (H.C.); parkhj@kmu.ac.kr (H.J.P.); 2 College of Pharmacy, Kyungsung University, Busan 48434, Republic of Korea; hlee@ks.ac.kr

**Keywords:** PROTAC, drug delivery system, physicochemical property, solubilization, bioavailability

## Abstract

Proteolysis targeting chimeras (PROTACs), heterobifunctional molecules that hijack the ubiquitin–proteasome system (UPS) to degrade specific proteins, hold great promise in treating diseases driven by traditionally “undruggable” targets. However, their large molecular weight, high hydrophobicity, and other physicochemical hurdles contribute to their limited bioavailability, suboptimal pharmacokinetics, and attenuated therapeutic efficacy. Consequently, diverse formulation innovations have been investigated to optimize PROTAC delivery. This review examines current challenges and advances in specialized drug delivery approaches designed to bolster PROTAC pharmacological performance. We first outline the fundamental limitations of PROTACs—their low aqueous solubility, poor cell permeability, rapid clearance, and concentration-dependent “hook effect”. We then discuss how various enabling formulations address these issues, including polymeric micelles, emulsions, amorphous solid dispersions, lipid-based nanoparticles, liposomes, and exosomes. Collectively, these delivery technologies substantially improve the therapeutic outcomes of PROTACs in preclinical cancer models. Future applications may extend beyond oncology to address other complex diseases using newly emerging heterobifunctional molecules. By integrating advanced formulation science with innovative degrader design, the field stands poised to unlock the clinical potential of PROTACs for protein degradation therapies.

## 1. Introduction

Proteins characterized by shallow binding pockets, broad active sites, or non-enzymatic scaffolding functions have historically been deemed “undruggable” by conventional small-molecule inhibitors [1]. Such structural and functional properties have hindered therapeutic success, leaving many disease-associated proteins out of reach. Despite the identification of over 4000 proteins implicated in various pathologies, only around 400 have been successfully targeted by approved treatments. This gap has driven the pursuit of next-generation therapeutics capable of overcoming these obstacles. Recently, proteolysis targeting chimeras (PROTACs) have emerged as a powerful approach to selectively eliminate disease-related proteins through the UPS [2,3,4].

**Figure 1:**
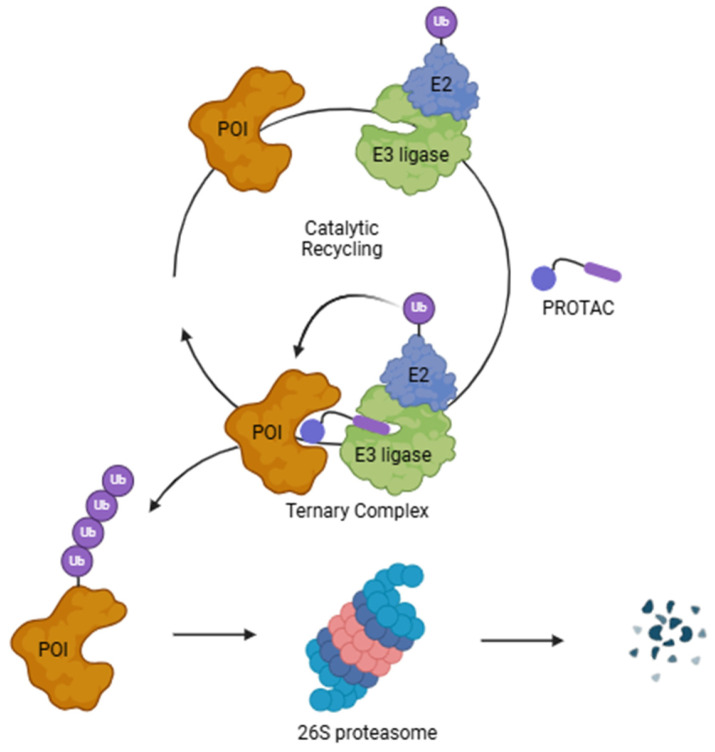
PROTAC’s mechanism of action.

PROTACs, heterobifunctional molecules that recruit a target protein to an E3 ubiquitin ligase, rely on reversible and non-covalent interactions to guide specific proteins toward proteasomal degradation, thereby reducing their functional level in disease models (Figure 1) [5]. Notably, following target proteolysis, PROTAC molecules dissociate and can participate in multiple rounds of protein degradation. This “recycling” feature underpins PROTACs’ heightened potency, reduced dosing, and durable activity. As a result, researchers are investigating PROTACs for proteins once considered undruggable, including SHP2, KRAS, Bcl-2, and p53, as well as for key transcription factors like STAT3, FoxM1, and c-Myc [6,7]. Furthermore, because PROTACs degrade proteins irrespective of their active sites, they can circumvent drug resistance triggered by target overexpression or mutation [7,8].

Interest in PROTACs has surged dramatically in recent years. According to the 2022 PROTAC database, 3260 PROTACs had been reported, and that number increased by 87% to 6111 in 2024 [9,10]. ARV-110 (NCT03888612) and ARV-471 (NCT04072952)—targeting the androgen (AR) and estrogen (ER) receptors, respectively—were the first to reach human trials in 2019, establishing proof of concept in 2020. As of 2025, a total of 15 PROTACs are being investigated in clinical studies (Table 1). Most PROTACs are in clinical phases I and II, and none have been approved yet for clinical application. However, the leading PROTACs, ARV-110 and ARV-471, have advanced to clinical phase III, showing their potential to be approved for clinical application soon. Yet, alongside this progress, practical hurdles persist—most notably the issue of low bioavailability due to their unfavorable physicochemical properties. For example, while cereblon (CRBN)-based PROTACs can often be administered orally, VHL-based PROTACs typically demand intravenous delivery and display lower bioavailability [11,12,13,14]. This discrepancy highlights a critical need for more effective PROTAC delivery platforms.

Achieving and maintaining sufficient therapeutic concentrations at the disease site constitutes another major hurdle for PROTAC therapy. Suboptimal drug distribution diminishes efficacy and increases off-target toxicity, thereby compromising clinical outcomes. Additional complications—such as potential chemical instability and ubiquitination of target proteins in non-target cells—can further undermine the stability and specificity of PROTACs. To overcome these barriers, advanced delivery technologies are essential. Over the past few decades, a range of drug delivery systems have been developed to enhance bioavailability, stability, and tissue selectivity for therapeutics with complex physicochemical properties. These systems can extend the circulation time of drugs, enable stimuli-responsive or targeted release, and minimize off-target disposition, collectively improving therapeutic outcomes [12,15,16].

Incorporating PROTACs into advanced delivery systems—through encapsulation, conjugation, or other approaches—can significantly enhance drug loading, protect the cargo from premature degradation, and precisely control drug release profiles at diseased sites [17,18]. These advances help address key challenges, including low solubility and the need for stable, prolonged systemic exposure of drugs. As the field evolves, the convergence of innovative PROTAC design with advanced drug delivery methodologies holds immense potential to reshape the therapeutic landscape of PROTACs for difficult-to-treat conditions.

Given the broad range of potential PROTAC architectures, it is necessary to define the focus of this review. Although numerous PROTAC modalities have been explored—from small-molecule- to peptide-based systems, this review centers predominantly on small-molecule PROTACs that have progressed into clinical evaluation. As evidenced by most ongoing clinical studies, small-molecule PROTACs represent mainstream development, highlighting their translational relevance and chemical adaptability [19,20]. Consequently, investigating specialized delivery systems for small-molecule PROTACs becomes pivotal for ensuring their clinical applicability and maximizing therapeutic outcomes. It also discusses various delivery strategies, such as solubilization technologies and chemical conjugation, aimed at optimizing therapeutic performance of PROTACs. For readers seeking a broader coverage of diverse PROTAC delivery platforms or earlier-stage discovery, we direct you to comprehensive reviews on these topics [21].

Building on these considerations, this review provides an in-depth examination of the latest advancements in specialized PROTAC delivery systems, detailing their design principles, benefits, and the challenges to clinical implementation. By highlighting key research efforts, we offer insights into emerging trends and the future trajectory of PROTAC-based therapies. Ultimately, the synergistic relationships between PROTAC innovation and advanced delivery strategies promises to open new frontiers in drug discovery, potentially revolutionizing treatments for a wide range of conditions [17].

## 2. Obstacles and Strategies for Optimal PROTAC Delivery

PROTACs offer a groundbreaking therapeutic strategy by hijacking the cells’ natural protein degradation machinery to efficiently eliminate disease-causing proteins. However, their therapeutic potential is significantly hampered by several challenges. These challenges include low aqueous solubility, poor cell permeability, off-target toxicity, and hook effects, which collectively limit their efficacy and clinical applicability [12]. Their low aqueous solubility stems from their complex molecular structure—comprising a ligand, warhead, and linker—and their high molecular weight, often referred as ‘molecular obesity’ [22,23]. This physicochemical property can impede the effective administration of the PROTACs and reduce absorption at the target site, ultimately leading to suboptimal therapeutic outcomes [24]. Additionally, the poor cell permeability of PROTACs, resulting from their high molecular weight and polar chemical surfaces with multiple hydrogen bond donors and acceptors, poses a significant barrier to their uptake by target cells [25]. This limitation hinders their ability to effectively engage the intracellular UPS and degrade proteins of interest, ultimately compromising their therapeutic performance. Furthermore, the concentration-dependent formation of complexes by PROTACs within the intracellular environment adds an extra layer of complexity in optimizing their therapeutic performance [26]. At higher concentrations, some PROTACs are more inclined to form binary complexes with either the E3 ligase or the target protein—rather than the desired ternary complexes—thereby reducing their efficacy [3]. This phenomenon is referred to as the “hook effect”, a hallmark of PROTAC pharmacology in which high concentrations favor the formation of non-productive binary complexes over functional ternary complexes. As a result, the degradation potency is diminished, creating another obstacle to achieving effective treatment. This phenomenon is encountered only in in vitro PROTAC studies and can easily mislead the assessment of their potential efficacy [27].

These limitations of PROTACs manifest as significant obstacles in both the parenteral and oral administration of PROTACs, highlighting the need for optimal delivery systems to achieve the desired therapeutic outcomes. For parenteral delivery, their poor solubility and permeability hinder systemic circulation, leading to suboptimal therapeutic outcomes. Also, PROTACs suffer from rapid renal clearance, necessitating frequent dosing and potentially complicating their pharmacokinetic profiles [17]. PROTAC efficacy via oral administration can be significantly hindered by unfavorable hurdles due to biological barriers of the gastrointestinal tract. The developability of these molecules is inherently challenging, as most PROTACs are classified as beyond the rule of five (Ro5), which correlates with diminished oral bioavailability. According to Ro5, poor absorption or permeation is more likely in compounds with more than five hydrogen bond donors (HBD > 5), a molecular weight exceeding 500 Da (MW > 500), and a calculated logP greater than five (cLogP > 5) [9,10]. In addition to being beyond Ro5, most PROTACs are categorized within the developability classification system as Class IIb or IV, indicating poor solubility and either good permeability (Class IIb) or poor permeability (Class IV). Their inherently low aqueous solubility predisposes them to aggregation within the gastrointestinal lumen, thereby hindering systemic absorption [28,29]. Furthermore, their extensive polar surface area impairs their membrane permeability in intestinal cells, potentially reducing their oral bioavailability [30,31]. Enzymatic degradation within the gastrointestinal tract may further limit their absorption into systemic circulation. Consequently, it is imperative to address these challenges for both parenteral and oral routes, as PROTACs must reach the cytosol of target cells to interact with their protein of interest (POI) and initiate proteasomal degradation [32]. Hence, devising effective delivery strategies for oral and injectable PROTAC formulations is crucial to fully harnessing their therapeutic potential.

Drug delivery systems present a promising approach to address the inherent challenges of PROTACs by improving their physicochemical properties, thereby fully realizing their therapeutic potential [33,34]. To address PROTAC challenges, various approaches have been developed, such as incorporating poorly soluble PROTACs into polymers, oils, or lipids within a delivery system, such as micelles, emulsions, and lipid nanoparticles. These delivery systems enhanced the pharmacological performance of PROTACs by improving their physicochemical properties, thereby fully realizing their therapeutic potential [33,34]. The following sections describe novel drug delivery strategies for PROTACs and analyze their impact on the physicochemical properties of PROTACs in preclinical models.

## 3. Delivery Systems for PROTACs

### 3.1. Polymeric Nanoparticle-Based Delivery Systems

Polymeric nanocarriers, which physically encapsulate or conjugate therapeutic agents to form polymeric nanomedicines, can self-assemble into a wide range of nanoscale architectures—including micelles, nanospheres, nanogels, polymersomes, and polymer–drug conjugates—when dispersed in aqueous media [35,36]. Various molecular interactions, such as hydrophobic interaction, hydrogen bonding, and, where relevant, electrostatic attractions between the polymer and the therapeutic agent, influence drug loading, stability, and controlled release [37,38]. In the context of poorly soluble small molecules, diblock or triblock copolymers with amphiphilic properties are generally exploited, incorporating both hydrophilic and hydrophobic domains to enable drug encapsulation and delivery [39,40,41]. A number of these polymers, including poly(lactic-co-glycolic acid) (PLGA), polyethylene glycol (PEG), polycaprolactone (PCL), poloxamers, and poly(ethylene oxide)-poly(propylene oxide)-poly(ethylene oxide) (PEO-PPO-PEO) copolymers, are well established in drug delivery applications due to their biocompatibility, biodegradability, and modifiability to control release kinetics or in vivo circulation [42,43,44]. Several recent studies have applied polymeric micelle systems to the delivery of PROTACs, demonstrating promising preclinical outcomes. By leveraging both the adaptable nature of polymeric carriers and their diverse molecular interactions, polymeric micelles can overcome challenges such as poor solubility, chemical instability, and suboptimal pharmacokinetic profiles inherent to PROTACs, strengthening their therapeutic efficacy.

Saraswat et al. developed a polymeric nanoparticle (ARV-NP) to enhance the delivery and therapeutic performance of ARV-825 to degrade the oncogenic c-Myc in pancreatic cancer [45]. Employing the nanoprecipitation method, they encapsulated ARV-825 using diblock PEG-*b*-PLGA (PEG–PLGA = 2 kDa:11.5 kDa), achieving a mean particle size of approximately 89 nm, a high encapsulation efficiency (~99%), and sustained drug release. Though the encapsulation efficiency was high, the loading capacity of ARV-825 was only 2% by weight due to the low mass ratio of the drug to excipients. The resultant ARV-NP formulation prolonged the half-life of ARV-825 by mitigating its enzymatic breakdown in in vitro liver microsomal assays, suggesting the protection of ARV-825 in the hydrophobic core of the polymeric nanoparticle using PEG-*b*-PLGA. Considering a high encapsulation efficiency and prolonged half-life of ARV-825, consequently, in vitro experiments further demonstrated that ARV-NP exerted greater cytotoxicity, induced more apoptosis, and more effectively suppressed migration and colony formation compared to the free drug. In a 3D pancreatic cancer model, ARV-NP reduced tumor spheroid viability, suggesting enhanced tumor penetration and prolonged drug action due to polymeric micelle formulation. Mechanistic studies revealed that ARV-NP effectively downregulated BRD4 and c-Myc expression while upregulating pro-apoptotic proteins like cleaved caspase-3, substantiating its role in targeted protein degradation and apoptosis induction. However, in vivo investigation is still lacking, and the in vivo performance of ARV-NP should be further explored in future studies. Taken together, polymeric micelle delivery systems demonstrated the potential to overcome the unfavorable physicochemical properties of ARV-825 and improve pharmacological performance.

The chemical versatility of polymers allows for additional modifications—such as ligand conjugation—to facilitate targeted drug delivery. By functionalizing these micelles with specific targeting ligands, the selectivity and therapeutic efficacy of PROTACs can further improve, enhancing the binding affinity and cellular uptake in diseased tissues. For example, Yang et al. developed a BRD4-targeting PROTAC nanodrug, termed SPP-ARV-825, for glioma therapy by employing a polymeric micelle platform designed to enhance blood–brain barrier (BBB) penetration and tumor targeting [46]. Substance P (SP) peptide was utilized as a targeting ligand for glioma, given its specific binding affinity to the overexpressed neurokinin 1 receptor in gliomas [47]. The formulation utilized SP-PEG-PDLLA and mPEG-PDLLA to encapsulate PROTAC ARV-825, yielding nanoparticles with an average size of 26.3 nm, uniform distribution, and controlled drug release over 96 h. An in vitro assay revealed that SPP-ARV-825 significantly inhibited glioma cell proliferation, induced G0/G1 cell cycle arrest, and triggered apoptosis through BRD4 degradation compared to free ARV-825. Moreover, the formulation better countered the immunosuppressive glioma microenvironment by reducing M2 macrophage polarization via the suppression of IRF4 transcription and diminished phosphorylation of STAT6, STAT3, and AKT, thereby undermining a key mechanism of glioma immune evasion. In vivo experiments confirmed robust tumor suppression by SPP-ARV-825 in both subcutaneous and orthotopic glioma models, marked by reduced tumor proliferation. By concurrently degrading BRD4 and mitigating immunosuppressive pathways, SPP-ARV-825 outperformed both free ARV-825 and non-targeted micelles, demonstrating the promise of brain-targeted polymeric micelle drugs in surmounting BBB constraints and effectively modulating the tumor microenvironment.

Polymeric micelles offer a versatile platform for co-encapsulating multiple therapeutic agents within a single particle, thus enabling the synchronized delivery of combination therapy. By loading two or more drugs together, these micelles can achieve spatial and temporal co-localization at the target site, which helps amplify therapeutic synergies [48,49]. Moreover, this co-delivery strategy can simplify treatment regimens by minimizing the number of separate injections or dosing schedules required for combination therapies [50,51]. Thus, the capacity for multimodal drug encapsulation within polymeric micelles holds significant promise for advancing combination therapies in cancer and other intractable diseases. Recent studies have also underscored the promise of combination therapy with PROTACs, preclinically demonstrating that co-delivering PROTACs alongside other agents can enhance the treatment efficacy and overcome drug resistance.

**Figure 2:**
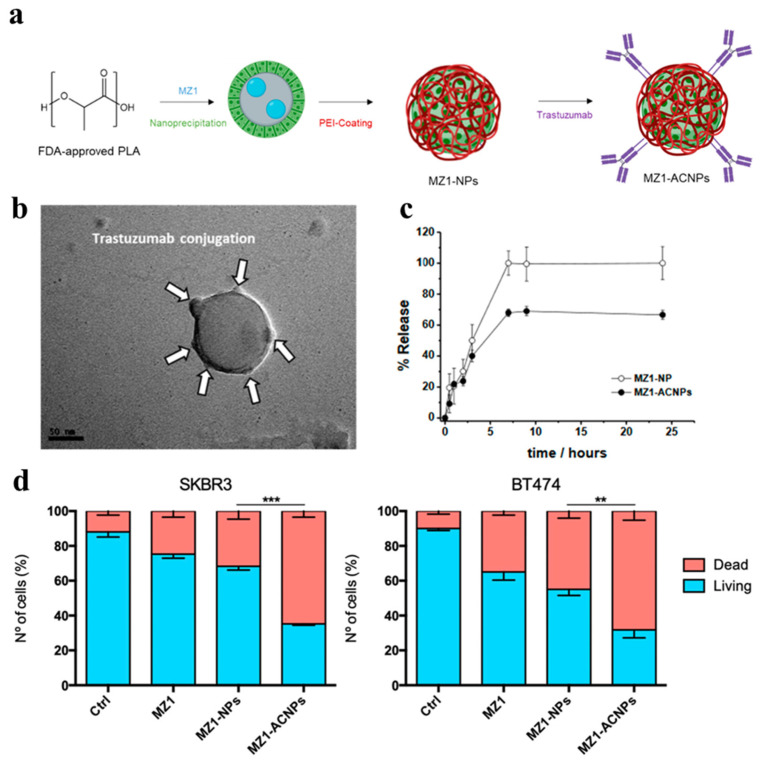
Preparation, characterization, and evaluation of polymeric antibody-conjugated nanoparticles. (**a**) Preparation of MZ1-loaded polymeric nanoparticles (MZ1-NPs) and MZ1-loaded polymeric antibody-conjugated nanoparticles (MZ1-ACNPs). (**b**) Transmission electron microscopy (TEM) images of MZ1-ACNPs. (**c**) In vitro drug release profiles of MZ1-NPs and MZ1-ACNPs in physiological conditions (pH 7.4). (**d**) Induction of apoptosis in HER2+ breast cancer cell lines (SKBR3 and BT474) after treatment with MZ1-ACNPs. (** *p* < 0.01, *** *p* < 0.001). Reproduced under the terms of the Creative Commons CC BY license from [52].

For instance, Cimas et al. investigated the development and characterization of MZ1-loaded polymeric antibody-conjugated nanoparticles (ACNPs) for targeted delivery in HER2-positive breast cancer (Figure 2) [52]. The nanoparticles are composed of FDA-approved polymers, polylactide (PLA), and polyethyleneimine (PEI), with PROTAC MZ1 encapsulated using nanoprecipitation techniques. Trastuzumab, an antibody targeting HER2 receptors, is conjugated to the nanoparticle surface via carbodiimide chemistry, enabling specific tumor targeting (Figure 2a). Characterization of the ACNPs revealed that the system had an average size of approximately 114 nm, with high stability and controlled drug release over 12 h (Figure 2b,c). These ACNPs exhibited enhanced in vitro cytotoxic effects and an increase in cell death by apoptosis against HER2-positive cancer cells (SKBR2 and BT474) compared to the free drug, showcasing their potential to overcome the physicochemical limitations of MZ1 and improve the drug delivery specificity to the target (Figure 2d). However, the in vivo efficacy of ACNPs has not been reported, necessitating further investigation in a preclinical animal model to evaluate the potential therapeutic benefits of combination therapy.

Chen et al. introduced a nanochaperone-based (nChap) mixed-shell polymeric micelle (MSPM) platform for the combined delivery of CXCL9 and the BRD4 PROTAC dBET6, effectively strengthening T cell-mediated antitumor immunity [53]. The MSPM was constructed using amphiphilic block copolymers, including PEG-*b*-PCL, COOH-PEG-*b*-PCL, and PCL-*b*-PAE, which self-assemble into a mixed-shell polymeric micelle. The system leverages hydrophobic PCL cores for encapsulating poorly soluble dBET6, while the hydrophilic PEG shell and carboxyl modification enable the electrostatic and hydrophobic loading of the protein CXCL9, maintaining its structural integrity. Within the tumor microenvironment, the formulation ensures controlled release of CXCL9, enhancing CD8+ T cell infiltration, while dBET6 triggers immunogenic cell death and inhibits IFNγ-induced PD-L1 expression to overcome immune evasion. Compared to administering free CXCL9 and dBET6, the nChap platform improves pharmacokinetics, increases tumor accumulation, reduces systemic toxicity, and extends circulation time. In vivo investigations show substantial tumor growth inhibition, prolonged survival, and superior therapeutic efficacy when used in combination with anti-PD-1 immune checkpoint blockade. The study demonstrated the potential of integrating chemokines and PROTACs in engineered nanoplatform to counteract immunosuppressive tumor environments and enhance cancer immunotherapy.

He et al. developed ARV-DOX/cRGD-P nanoparticles for targeted colorectal cancer therapy [54]. This system employs a cyclo(Arg-Gly-Asp-D-Phe-Lys) (cRGD)-modified PEG-*b*-PCL block copolymer to co-deliver doxorubicin (DOX) and BRD4 PROTAC degrader ARV-825. Produced via a self-assembly, resulting core–shell features a PEG-based outer layer for hydrophilicity, while the cRGD ligand facilitates selective binding to integrin αvβ3 on tumor cells. The researchers achieved high drug loading (DL) and encapsulation efficiency (EE): in ARV-DOX/cRGD-P, DOX had a DL of 2.5% and an EE of 94%, while ARV-825 had a DL of 5% and an EE of 98.4%. The nanoparticles demonstrated stability, retained a small particle size (~59 nm), and produced a synergistic antitumor effect in animal models. By integrating passive and active targeting strategies, this design enhances the therapeutic efficacy of PROTAC-based combination therapy and reduces systemic toxicity.

Environment-responsive polymeric micelles have emerged as adaptive carriers for delivering PROTACs, leveraging their ability to respond to pH, temperature, or redox changes in the tumor microenvironment. Such dynamic behavior ensures precise spatiotemporal drug release and higher target specificity, while the inherent chemical versatility of polymers enables further modifications for enhanced ligand-mediated uptake and minimized off-target effects [55,56].

**Figure 3:**
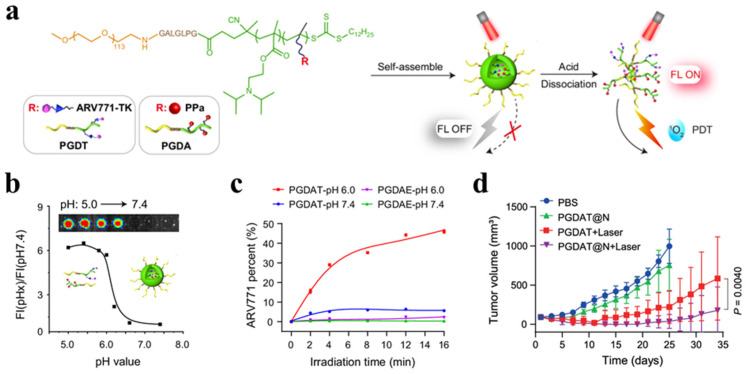
An ROS/hypoxia dual-activatable region-confined PROTAC nanoparticle. (**a**) Formation of PGDAT nanoparticle through self-assembly with acid-activatable and ROS-responsive properties. (**b**) Acid-induced disaggregation of the PGDAT nanoparticle and fluorescence imaging of nanoparticles at different pH values. The fluorescence indicates the acid-induced disaggregation of the PGDAT nanoparticles. (**c**) Acid-triggered photoactivation and drug release profile of PROTAC nanoparticles under laser irradiation at pH 7.4 and 6.0 (ROS-sensitive, PGDAT; ROS-inert, PGDAE). (**d**) Tumor progression inhibition after PGDAT nanoparticle treatment with laser irradiation in TNBC models. Reproduced with permission from [57]; published by Springer Nature, 2024.

Gao et al. developed a reactive oxygen species (ROS)-activatable and hypoxia-responsive PROTAC prodrug by incorporating BRD4 PROTAC ARV-771, with a thioketal group as an ROS-responsive polymer and a nitrobenzyl group as a hypoxia-responsive polymer (Figure 3a) [57]. These components were then encapsulated into a self-assembling PROTAC nanoparticle (PGDAT) system. By utilizing the unique characteristics of the tumor microenvironment, this region-confined PROTAC system enhanced the degradation of BRD4 and amplified the antitumor efficacy of ARV-771, which is activated by the stimuli-responsive system within tumor cells (Figure 3b,c). Moreover, the system demonstrated improved accumulation and penetration at the tumor site, enabling targeted PROTAC delivery. Notably, this region-confined PROTAC system was effective in both normoxic and hypoxic environments, resulting in significant tumor progression inhibition in triple-negative breast cancer (TNBC) and head-and-neck tumor models (Figure 3d). These findings underscore the potential of stimuli-responsive delivery systems to efficiently deliver PROTACs, offering a promising strategy for targeted cancer therapy.

Liu et al. presented a glutathione-scavenging polymeric nanoparticle-mediated PROTAC delivery system to enhance drug exposure and therapeutic efficacy [58]. The ARV@PDSA Nano-PROTACs, constructed using poly(disulfide amide) (PDSA) polymeric nanoparticles, encapsulated BRD4 degrader ARV-771, providing redox-responsive drug release that enhances intracellular accumulation and targeted protein degradation. The formulation scavenged glutathione (GSH), reinforcing BRD4 degradation and inducing c-Myc-related ferroptosis and cell cycle arrest, leading to superior antitumor effects in vitro and in vivo compared to those of free ARV-771. The polymeric nanoparticle exhibited prolonged blood circulation and enhanced tumor penetration, effectively increasing BRD4 degradation and reducing the tumor burden in both xenograft and syngeneic mouse models. This approach showcased an environment-responsive polymeric micelle strategy to enhance the exposure of PROTACs to disease targets and enhance therapeutic outcomes in cancer treatment.

Zhang et al. introduced CREATE, an innovative nano-PROTAC delivery platform designed to improve the efficacy and specificity of BRD4 PROTAC dBET6 for lung cancer therapy [59]. dBET6 was encapsulated in pH- and glutathione-responsive, disulfide-linked poly (lactic-co-glycolic acid) (DS-PLGA) nanoparticles, enabling controlled release in the tumor microenvironment. To enhance tumor targeting, these nanoparticles were camouflaged with engineered lung cancer cell membranes (CRV-LLCM), incorporating a CRV peptide for selective binding to both lung cancer cells and tumor-associated macrophages (TAMs). This dual-targeting mechanism increases intratumoral accumulation and promotes caveolin-mediated endocytosis, ensuring efficient intracellular uptake. Under acidic and reductive tumor conditions, the DS-PLGA polymer degrades to release dBET6 more effectively within cancer cells. An in vivo investigation demonstrated the superior tumor growth inhibition of CREATE to that of the free drug, attributed to its capacity to simultaneously target cancer cells and immunosuppressive TAMs, thereby remodeling the tumor microenvironment.

Guan et al. developed a dual-targeting, bioresponsive nano-PROTAC system to improve the therapeutic performance of BRD4-targeting PROTAC dBET6 in lung cancer. This formulation leverages a pH- and glutathione-responsive polymer (DS-PLGA) for controlled intracellular release, while the lung cancer cell membrane (LLCM) coating and cRGD ligands collectively provide homotypic and active tumor targeting. Characterization confirmed the nanoparticles’ stability, high drug-loading capacity, and triggerable drug release under acidic and reducing conditions, resulting in enhanced BRD4 degradation, caspase-3 activation, and apoptosis in lung cancer cells. In vivo, the system achieved prolonged circulation, elevated tumor accumulation, and substantial tumor suppression compared to free dBET6 or non-targeted formulations, all with minimal systemic toxicity. Overall, this bioinspired nano-PROTAC strategy demonstrates how integrating tumor-specific targeting and stimulus-responsiveness can overcome the conventional challenges of PROTAC solubility, bioavailability, and off-target effects [60].

**Figure 4:**
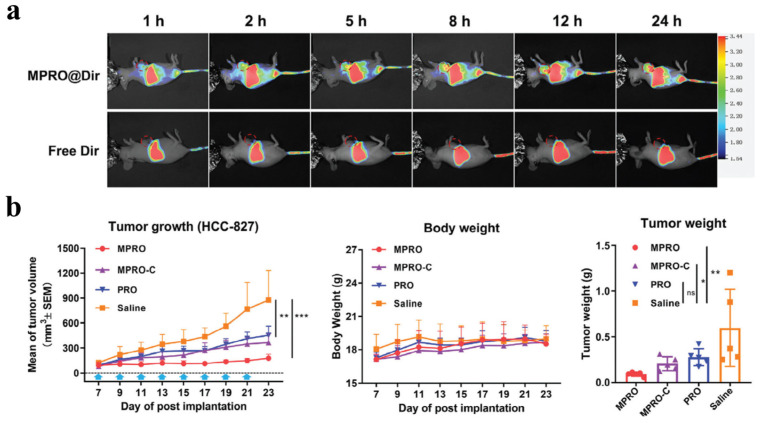
Evaluation of folate-PEG-PROTAC micelles (MPRO). (**a**) The biodistribution of MPRO@Dir and free Dir in HCC-827-bearing nude mice after intravenous administration at 1, 2, 5, 8, 12, and 24 h. (**b**) In vivo antitumor efficacy and toxicity in HCC-827-bearing nude mice. (* *p* < 0.05, ** *p* < 0.01, *** *p* < 0.001 versus the Saline group). Reproduced with permission from [61]; published by John Wiley and Sons, 2024.

Ma et al. introduced folate-PEG-PROTAC micelles (MPRO) as tumor-targeted nanocarriers designed to improve the bioavailability, specificity, and overall efficacy of PROTAC (Figure 4) [61]. By conjugating an EGFR-degrading PROTAC to folic acid–polyethylene glycol (FA-PEG) via a GSH-responsive disulfide bond, MPRO exploits folate receptor overexpression on tumor cells for selective internalization. These micelles self-assemble into stable nanoparticles (~50 nm) with enhanced water solubility, extended circulation time, and increased tumor accumulation through receptor-mediated endocytosis. Upon cellular uptake, the GSH-cleavable linkage breaks, releasing the active PROTAC to degrade EGFR and trigger tumor cell apoptosis. In vitro studies revealed significantly improved cellular uptake, robust EGFR degradation, and stronger cytotoxic effects against HCC-827 and PC-9 lung cancer cells compared to the free PROTAC. In vivo, MPRO achieved higher tumor accumulation (Figure 4a), minimized off-target toxicity, and a 79.6% tumor growth-inhibition rate, outperforming both the free PROTAC and a non-cleavable control micelle (Figure 4b).

X-ray radiation therapy is a primary clinical treatment for cancer. It works by using high-energy X-rays to destroy cancer cells and shrink tumors, making it an essential component of modern oncology [62]. Xu et al. developed an X-ray radiation-responsive PROTAC nanomicelle (RCNprotac) to enhance radiotherapy [63]. The design involved linking PEG to BRD4 PROTAC MZ1 through a diselenide bond, resulting in the self-assembly of a nanomicelle with an average size of 141.80 ± 5.66 nm. The RCNprotac remained inactive during circulation, as the hydroxyl group on the E3 ubiquitin ligase component was occupied, ensuring stability. It accumulated efficiently at tumor sites due to the enhanced permeability and retention (EPR) effect. When exposed to X-ray radiation, the diselenide bonds were broken, releasing MZ1 specifically at the tumor site to degrade BRD4 proteins. This targeted approach boosted antitumor efficacy both in vitro and in vivo. The X-ray-responsive PROTAC nanomicelle represented a novel approach for achieving precise, X-ray-triggered protein degradation and enhancing tumor sensitivity to radiotherapy through BRD4 proteolysis.

As discussed above, polymeric nanocarriers serve as versatile platforms capable of encapsulating a wide range of therapeutic agents, including PROTACs, thereby improving solubility, enhancing stability, and enabling controlled release. Through self-assembly, these carriers can encapsulate either single or combination therapeutics, as evidenced by numerous preclinical studies demonstrating improved tumor accumulation, enhanced therapeutic efficacy, and reduced systemic toxicity. These findings highlight the promise of polymeric micelle-based strategies for optimizing PROTAC therapy. Despite the progress in polymeric nanoparticle-based delivery systems, several challenges remain. Two major challenges are achieving the ideal polymer-to-drug ratio for stable formulation and maintaining particle size stability. Additionally, concerns regarding toxicity due to unexpected immunogenic reactions of polymers and an increased risk of particle aggregation in vivo may further complicate their application.

### 3.2. Emulsion-Based Delivery Systems

Emulsion-based drug delivery systems have been extensively investigated for improving the delivery of poorly water-soluble molecules, offering enhanced solubility, stability, and permeability. By employing these systems, many compounds with low aqueous solubility have become viable candidates for both systemic and oral administration [64,65]. A microemulsion is an isotropic system, generally consisting of a drug, oil, surfactant, co-surfactant, and even a co-solvent that is thermodynamically stable [65,66]. When designing such formulations, the choice of oils, surfactants, co-surfactants, and co-solvents is of importance [66]. Commonly used excipients include pharmaceutical oils, non-ionic surfactants (e.g., polysorbates such as Tween 80) for emulsification, and co-surfactants like propylene glycol or PEG to further improve stability and drug-loading capacity [67,68]. Key physicochemical parameters—such as droplet size, zeta potential, polydispersity index, and viscosity—play an important role in determining the in vitro and in vivo performance of emulsion formulation, influencing factors such as drug release kinetics, bioavailability, and emulsion drug stability. A notable innovation within this domain is the self-nanoemulsifying drug delivery system (SNEDDS), which spontaneously generates fine oil-in-water emulsions upon contact with aqueous environments (e.g., gastrointestinal fluids) [69]. SNEDDS confers several advantages, including increased drug solubility and dissolution rates, improved bioavailability stemming from a reduced particle size and higher surface area, and protection against degradation in the gastrointestinal tract [70,71]. These formulations are typically optimized by adjusting the ratio of oil, surfactant, and co-surfactant to maximize drug solubility and stability within the system [72,73,74].

A recent study by Rathod et al. reported that PROTAC-loaded SNEDDS improved the PROTAC uptake in target cells and therapeutic performances [75]. This study investigated the anticancer activity enhancement of BRD4 PROTAC ARV-825, using an emulsion-based formulation in both vemurafenib-sensitive and -resistant melanoma cells. To address ARV-825’s poor aqueous solubility (<7 µg/mL) and pH-dependent degradation, an ARV-825-loaded self-nanoemulsifying preconcentrate (ARV-SNEP) was developed with dimethyl acetamide, medium chain triglycerides, and Kolliphor ELP. The ARV-SNEP formed nanoglobules (45.02 nm, −3.78 mV zeta potential) and significantly improved ARV-825’s solubility by nearly 66-fold and 300-fold in fed- and fasted-state simulated gastric fluid, respectively, without any precipitation of the drug. The authors also found that ARV-825 is a CYP3A4 substrate but not a P-gp efflux pump substrate, suggesting potential combination therapy with CYP3A4 inhibitors for oral delivery of ARV-825. ARV-SNEP exhibited superior anticancer efficacy, anti-migration, and pro-apoptotic effects over free ARV-825 in BRAF inhibitor-resistant melanoma cells at nanomolar drug concentrations. This highlights the importance of optimal formulation design and improved physicochemical properties for enhanced therapeutic potential of PROTAC drugs.

Saraswat et al. developed an emulsion-based combination therapy system that facilitates the oral delivery of both ARV-825 (PROTAC) and vemurafenib (BRAF inhibitor). ARV-825 effectively downregulates c-Myc expression, which resensitizes melanoma cells that are resistant to BRAF inhibitors, thereby producing synergistic cytotoxic, anti-migratory, and pro-apoptotic effects [76]. To further optimize therapeutic efficacy, the researchers formulated oral lipid nanocomplexes—referred to as NANOVB—that encapsulate both ARV-825 and vemurafenib, improving the aqueous solubility and in vitro permeability of the drugs. The in vivo experiments demonstrated that NANOVB markedly inhibited tumor growth, extended the survival in mice, and reduced the expression of BRD4 and Ki-67 proteins. These results underscore the effectiveness of emulsion-based drug delivery systems in enhancing the performance of PROTACs by improving drugs’ aqueous solubility and permeability.

As discussed above, emulsion-based drug delivery systems, particularly self-nanoemulsifying formulations, represent an innovative strategy for improving the solubility, stability, and permeability of poorly water-soluble PROTAC molecules. Recent studies on PROTAC-loaded SNEDDS and emulsion-based combination therapies underscore the capacity of these systems to not only enhance therapeutic outcomes but also overcome drug resistance by enabling synergistic treatments. Overall, the potential of these emulsion-based approaches emphasizes the need for formulation design in unlocking the full therapeutic potential of PROTACs. However, the stability and release profile of these systems may be dependent on the selection of oils, surfactants, and co-surfactants, making it difficult to find an optimal formulation. Additionally, the inclusion of certain excipients, such as dimethylacetamide used in the formulation of PROTAC-loaded SNEDDS by Rathod et al., may raise potential excipient-derived toxicity. Furthermore, similar to other emulsion-based systems, SNEDDS formulations face challenges like drug precipitation and formulation instability, characterized by a tendency to phase separation over time. These issues may complicate both large-scale production and long-term storage of emulsion formulations of PROTACs.

### 3.3. Solid Dispersion-Based Delivery Systems

Amorphous solid dispersions (ASDs) are among the most commonly employed techniques to enhance the solubility of drugs with poor aqueous solubility [77]. By generating and maintaining supersaturated concentrations of the drug in solution, ASDs can significantly enhance dissolution rates and consequently increase in vivo absorption [78]. Central to the development of ASD formulations is the careful selection of polymeric carriers—such as polyvinylpyrrolidone (PVP), hydroxypropyl methylcellulose, or Soluplus^®^—to ensure both the physicochemical stability of ASDs and sufficient drug loading into ASDs [79,80]. Optimizing the drug-to-polymer ratio thus plays a crucial role in preventing recrystallization and preserving the amorphous state, while manufacturing processes such as spray-drying and hot melt extrusion are often employed to create a uniform, thermodynamically stable dispersion [81]. In addition to these core considerations, factors such as polymer–drug compatibility, processing conditions, and downstream formulation steps can influence the pharmaceutical performance of ASD drugs [78]. Notably, several recent studies have explored the potential of ASDs to improve the solubility of PROTACs, thereby enhancing the drug efficacy in preclinical models.

Pöstges et al. examined the use of ASD formulations to overcome oral delivery challenges associated with AR PROTAC ARCC-4 [82]. Through supersaturation screening, hydroxypropyl methylcellulose acetate succinate and Eudragit^®^ L 100-55 were selected from multiple polymers, and the resulting ASD formulation significantly boosted the aqueous ARCC-4 solubility while maintaining a prolonged supersaturated state in aqueous media. In contrast, liquisolid systems prepared using biocompatible organic solvents and mesoporous silica gel failed to enhance drug dissolution, despite an observed increase in ARCC-4 solubility, underscoring the importance of polymer–drug interactions in stabilizing supersaturation. Their findings suggest that ASDs hold promise for addressing the poor solubility of ARCC-4, though additional research is warranted to resolve the inherent permeability issues of PROTACs in vivo. On the other hand, Mareczek et al. explored polyvinyl alcohol (PVA)-based spray-dried dispersions in improving the solubility and stability of the ARV-110 and SelDeg51 [83]. By embedding these PROTACs in PVA matrices using a three-fluid nozzle spray-drying technique, the authors achieved significant solubility enhancements for both crystalline (ARV-110) and amorphous (SelDeg51) PROTACs, along with stable amorphous solid dispersions over four weeks. The activity assays confirmed that the bioactivity and protein degradation capacity of SelDeg51 remained intact after the spray-drying process. These results underscore the potential of polymer-based ASDs as an effective strategy to address the solubility and challenges of PROTAC drugs.

**Figure 5:**
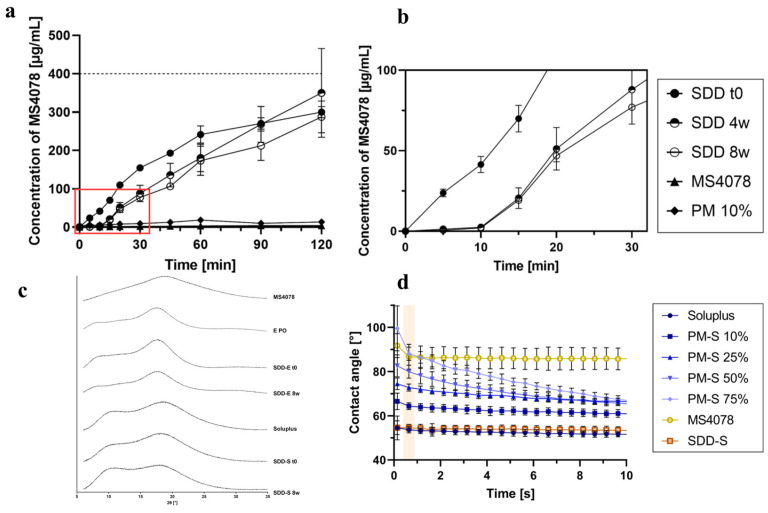
Evaluation and stability of spray-dried dispersion (SDD). (**a**) Dissolution profiles of the SDDs, API (MS4078), and physical mixtures (PM) in fasted-state simulated intestinal fluid and after 4 and 8 weeks at 40 °C/75% RH. (**b**) Magnified view of the first 30 min (**a**) of the dissolution profiles. (**c**) X-ray diffraction (XRD) patterns of API, excipients, and SDD initially and after 8 weeks at 40 °C/75% RH. (**d**) Wettability analysis using contact angle measurement. Reproduced under the terms of the Creative Commons CC BY license from [84].

Hofmann et al. explored the use of ASDs via spray-drying to improve the solubility and dissolution properties of model PROTAC MS4078 (Figure 5) [84]. Through systematic excipient screening, Soluplus^®^ and Eudragit^®^ E PO emerged as suitable polymers, demonstrating strong supersaturation stabilization in biorelevant media (Figure 5a). Spray-dried dispersions of the MS4078 attained over a 70-fold increase in supersaturation relative to the crude amorphous active pharmaceutical ingredient (API), significantly outperforming the corresponding physical mixtures (Figure 5b). Advanced characterization using energy-dispersive X-ray spectroscopy and Raman imaging confirmed the uniform API distribution within the polymer matrix, thereby improving the dissolution kinetics (Figure 5c). Further wettability analysis revealed that Soluplus-based solid dispersion of MS4078 enhanced surface wetting, promoting rapid dissolution, while the particle size influenced the dissolution rates but not the supersaturation stability (Figure 5d). Although the spray-dried dispersions remained physically stable in dry environments for at least eight weeks, high humidity led to degradation, underscoring the necessity of effective moisture protection.

Taken together, these findings suggest that ASDs may improve the solubility, stability, and potential oral bioavailability of PROTACs, thereby broadening the therapeutic applicability of PROTAC drugs. Despite their ability to enhance the solubility of PROTACs, all the PROTAC-ASD systems discussed above lack in vivo data. Many ASD-based delivery systems for PROTACs remain in the early stages of development, limiting the availability of clinical pharmacokinetic data. Additionally, the diversity of polymers that can be used, along with the various manufacturing methods, requires a case-by-case evaluation in vivo. Furthermore, challenges such as recrystallization and maintaining supersaturation are significant barriers to large-scale production.

### 3.4. Lipid Nanoparticle-Bassed Delivery Systems

Lipid nanoparticles (LNPs) are versatile nanoscale lipid-based carriers increasingly utilized in pharmaceutical research [85,86]. Several drugs with lipid-based delivery formulations have received regulatory approval from the FDA, primarily for the treatment of cancer and its associated complications [87]. They typically incorporate various lipids—such as phospholipids, solid lipids, or ionizable lipids—along with stabilizers like cholesterol or PEGylated lipids. In contrast to liposomes, which consist of one or more phospholipid bilayers, LNPs often feature a solid or semi-solid lipid core in the nanoparticle. This architectural difference broadens their capacity for encapsulating diverse therapeutic cargo, including small-molecule drugs, nucleic acids, and proteins [88,89]. Recent investigations on drug delivery using LNP formulations have demonstrated the improvement in drug stability, loading capacity, and the membrane permeability towards drug targets [90,91,92]. As a result, LNPs play a pivotal role in drug delivery strategies such as gene therapy, vaccine development, and targeted drug delivery. Several studies have reported LNP-based strategies for PROTAC delivery, primarily to improve the membrane permeability and promote efficient cytosolic proteolysis.

The study by Vartak et al. exploited a nanostructured lipid carrier (NLC) for delivering ARV-825 in in vitro lung carcinoma treatment [93]. Given the solubility challenges of the ARV-825, the authors developed ARV-825-loaded PEGylated NLCs (AP-NLC) using melt emulsification, stabilizing ARV-825 with Precirol^®^ ATO5, Captex^®^ 300, and decanoic acid. The resulting AP-NLC demonstrated a hydrodynamic diameter of ~56 nm, high drug encapsulation efficiency, and increased drug stability against metabolic degradation. In vitro studies demonstrated that AP-NLC effectively inhibited lung cancer cell migration, colony formation, and proliferation. It also enhanced BRD4 degradation, c-Myc suppression, and apoptosis while exhibiting minimal hemolytic toxicity, supporting its potential for systemic administration. Overall, this study highlights the potential of lipid-based nanocarriers to enhance PROTAC drug delivery for improved lung cancer therapy.

**Figure 6:**
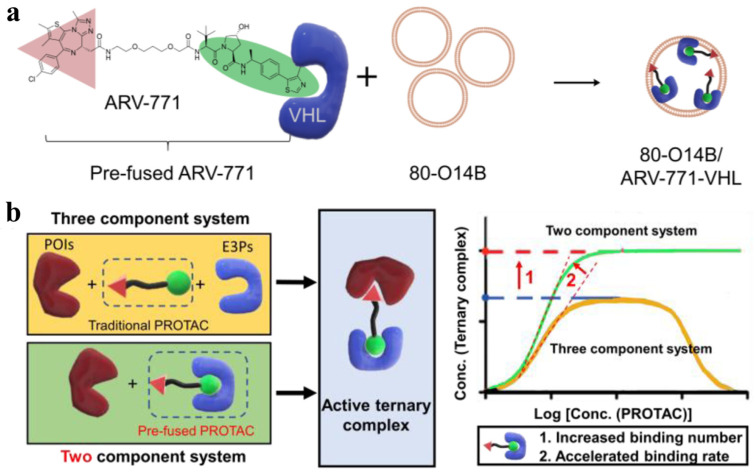
Preparation and protein degradation of pre-fused PROTAC-loaded LNP. (**a**) The preparation of pre-fused ARV-771-loaded 80-O14B LNP. (**b**) Formation of active ternary complex and binding amount and rate of two or three component system. Reproduced with permission from [94]; published by Elsevier, 2021.

Chen et al. introduced an innovative formulation strategy to enhance intracellular delivery and efficacy of PROTAC by encapsulating “pre-fused” PROTACs in LNPs (Figure 6) [94]. By fusing PROTAC molecules with their corresponding E3 ligase-related proteins prior to administration, they effectively converted the conventional three-component system into a more streamlined two-component system, thereby accelerating target protein degradation. Using synthetic lipid 80-O14B, the optimized LNP formulation significantly improved cellular uptake and retention of the pre-fused PROTACs, as evidenced by an increase in the encapsulation efficiency from 27.2% to 60.8%. In vitro studies demonstrated that ARV-771-loaded LNPs achieved over a 90% reduction in BRD4 levels within 24 h—far surpassing the modest degradation observed with free PROTACs—while also halving BRD4 expression within just 6 h, a process that took 24 h with conventional PROTACs. The applicability of this strategy was further confirmed with SARD279, wherein pre-fusion to heat shock protein 70 yielded the 80% degradation of the AR at substantially lower concentrations. Overall, this approach highlights the potential of combining pre-fusion methodologies with lipid-based nanocarriers to overcome PROTAC delivery challenges, offering enhanced protein degradation kinetics.

Saraswat et al. designed a dual lipid nanocarrier approach to co-deliver a PTEN plasmid and the BRD4-targeting PROTAC (ARV-825) for synergistic suppression of c-Myc-driven, drug-resistant melanoma [95]. Based on the distinct physicochemical characteristics of these two therapeutic agents, they prepared separate formulations—PTEN plasmid-loaded lipid nanoparticles (PL-NANO) and ARV-825-loaded nanoliposomes (AL-NANO)—achieving sub-100 nm particle sizes and encapsulation efficiencies over 99%. The combined delivery of PL-NANO and AL-NANO enhanced cytotoxicity in both 2D and 3D in vitro melanoma models, leading to marked apoptosis and tumor growth inhibition. Mechanistic studies revealed that this co-formulation upregulated PTEN levels, degraded BRD4, and ultimately downregulated c-Myc to curb the proliferation of BRAF inhibitor (BRAFi)-resistant melanoma cells. Notably, the IC_50_ values for ARV-825 were reduced by as much as 14.4-fold, indicating the potential for lower dosing and improved safety. Additional assays for migration and vasculogenic mimicry confirmed that the dual nanocarrier system effectively impeded tumor invasion and metastasis, while 3D spheroid models showed the significant inhibition of tumor expansion.

Overall, LNPs stand out as versatile platforms for delivering PROTACs, addressing key challenges such as poor solubility and limited stability. Notably, LNPs can traverse physiological barriers and facilitate cellular uptake through multiple mechanisms, thereby improving the suboptimal membrane permeability of PROTACs. These findings collectively underscore the promise of lipid-based delivery strategies in expanding the therapeutic window for PROTACs and safer cancer treatments. However, due to the current lack of in vivo preclinical studies of PROTAC-loaded LNP formulations, further evaluation in animal models is necessary to fully characterize the drug delivery profile and confirm improvements in the PROTAC efficacy by LNP formulations.

### 3.5. Liposome-Based Delivery Systems

Liposomal encapsulation is a widely used strategy to improve the solubility, permeability, and efficacy of poorly soluble small drugs. This approach offers several benefits, including solubilization, sustained/controlled release, protection against degradation and clearance, enhanced therapeutic efficacy, and reduced toxicity [96,97]. Liposomes are spherical vesicles formed by one or more phospholipid bilayers and can range from tens to hundreds of nanometers in size, enabling the encapsulation of both hydrophilic and hydrophobic drugs [98,99]. The lipid composition, fluidity, and surface charge are tunable, impacting the stability, circulation time, and drug release profiles [100]. Drug loading can occur passively during liposome formation or actively through techniques leveraging ion gradients or pH differences, leading to efficient encapsulation [101]. Liposomes are usually manufactured using established methods such as thin-film hydration, reverse-phase evaporation, and microfluidics, which allow for scalable, reproducible production [102]. Recently, liposome-based drug delivery systems have been applied to deliver PROTACs to enhance their solubility, permeability, and overall efficacy.

Zhang et al. developed a liposomal formulation (DT@NPs) that encapsulates DT2216—a PROTAC targeting Bcl-xL—to address challenges related to solubility, bioavailability, and tumor specificity [103]. Using a thin-film hydration method, they produced stable nanoliposomes with an average diameter of approximately 100 nm, which promotes prolonged circulation and higher tumor accumulation. In comparison to free DT2216, DT@NPs showed superior uptake by triple-negative breast cancer (MDA-MB-231) cells, resulting in greater Bcl-xL degradation and notably elevated cytotoxic effects. The in vitro data revealed that DT@NPs induced higher rates of apoptosis, stronger inhibition of colony formation, and reduced cell migration relative to the free drug. Animal studies confirmed that this nanoliposomal approach produced more substantial tumor suppression, enhanced biodistribution, and improved tumor selectivity. Notably, the formulation did not induce significant systemic toxicity or thrombocytopenia, effectively mitigating a primary safety concern associated with Bcl-xL-targeted treatments. Collectively, these findings underscore the potential of nanoliposomal delivery in enhancing the efficacy and safety of PROTAC-based cancer therapies.

Liposome-based drug delivery systems decorated with targeting ligands offer a promising strategy for precise, site-specific therapy. By conjugating ligands that bind selectively to overexpressed receptors on diseased cells, these formulations can achieve enhanced accumulation at the target site. This targeted approach capitalizes on the inherent benefits of liposomes, including drug solubilization, protection, and controlled release, while providing active specificity and improved efficacy. Research from Saraswat et al. reported a new galactose-decorated nanoliposomal formulation (GALARV) specifically designed to deliver ARV-825 better to hepatocellular carcinoma (HCC) cells [104]. By targeting the asialoglycoprotein receptor overexpressed on these cancer cells, GALARV showed a 4.5-fold increase in drug uptake compared to non-targeted liposomes. It exhibited a favorable nanometer-scale size (approximately 93 nm), high encapsulation efficiency (around 99%), and long-term stability for up to six months, thus facilitating drug delivery and efficacy. GALARV significantly elevates the intracellular levels of ARV-825, enhancing BRD4 degradation and subsequently suppressing the oncogenic c-Myc pathway, resulting in potent apoptotic effects. Comparative in vitro cytotoxicity assessments reveal that GALARV achieves a markedly lower IC_50_ value than free ARV-825, indicating superior drug delivery and efficacy. The formulation also shows improved penetration into 3D liver cancer spheroids, leading to notable tumor suppression and underscoring its potential in vivo applications.

Liposomal combination drug delivery harnesses the synergy of co-encapsulated therapeutics to enhance the therapeutic performance of drugs. By incorporating multiple agents within a single liposomal carrier, these systems can coordinate drug release at the target site, optimizing drug exposure. Moreover, the lipid composition, fluidity, and surface charge of the liposomes can be tailored to maximize stability and enable the controlled, sustained release of multiple payloads. Zhang et al. proposed a drug combination strategy by encapsulating DT2216 within a nanoliposomal system. This system was further enhanced by binding siRNA to the surface of the liposomes, followed by the addition of fluorinated polyethyleneimine to protect the siRNA and facilitate endocytosis and endosomal escape. With an approximate size of 200–300 nm, this system significantly improved the solubility of DT2216, one of the most insoluble PROTACs. Moreover, it effectively degraded target proteins and inhibited tumor cells in the H1299 cell line, demonstrating potent efficacy both in vivo and in vitro. This approach highlighted the potential to enhance the therapeutic efficacy of PROTACs while overcoming challenges such as drug resistance and poor solubility [105].

The paper from Fu et al. reported a novel PEGylated nanoliposomal formulation that co-delivers a protein kinase C inhibitor and a BRD4 PROTAC, ARV-825, to address vemurafenib-resistant melanoma [106]. Palmitoyl-DL-carnitine chloride imparted a positive charge on the liposomes, enhancing their stability and controlled drug release. This formulation exhibited nanoparticles of approximately 105 nm in diameter, with a significantly positive zeta potential (+26.6 mV) that facilitates higher uptake by melanoma cells. Comparative cytotoxicity assays revealed that the nanoliposome formulation outperformed single-drug treatments, evidenced by a substantially lower IC_50_. In addition, anti-angiogenesis and anti-vasculogenic mimicry studies confirm its effectiveness in inhibiting endothelial tube formation and melanoma cell migration. Overall, the study highlighted liposomal formulations for the delivery of PROTAC as a promising strategy for overcoming drug resistance and improving therapeutic outcomes in cancer.

**Figure 7:**
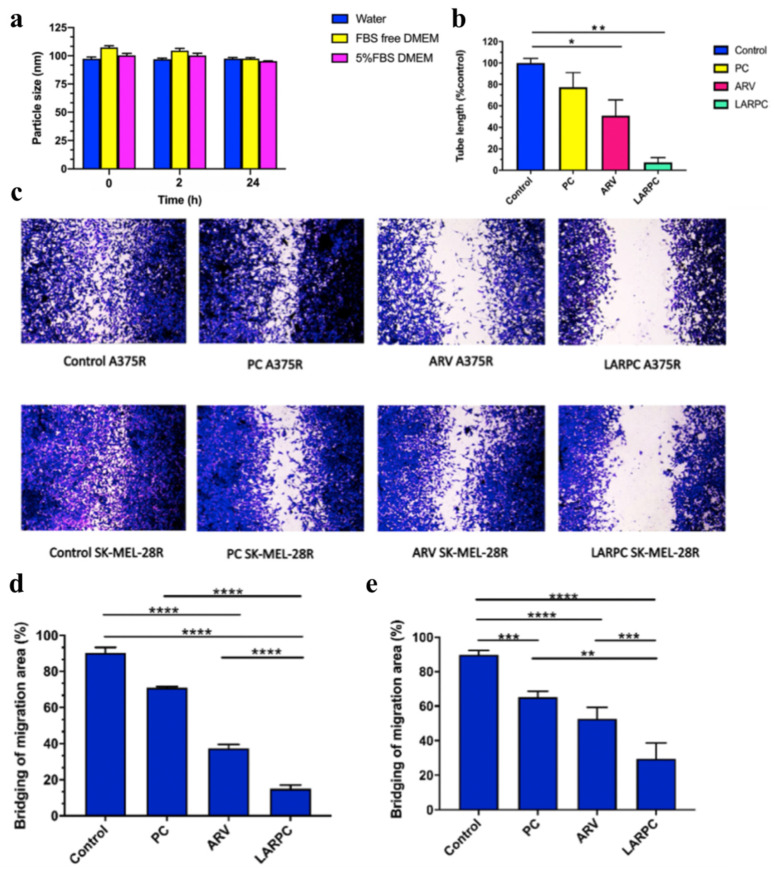
Characterization and evaluation of ARV-loaded PEGylated nanoliposomes (LARPC). (**a**) The particle size of LARPC in various media. (**b**) In vitro anti-angiogenesis assay based on tube formation. In vitro migration inhibition assay by scratch-wound method in melanoma cell lines. (**c**) Microscopic images with crystal violet (×10 magnification). Scratch bridging in (**d**) A375R and (**e**) SK-MEL-28 R cell lines. (* *p* < 0.05, ** *p* < 0.01, *** *p* < 0.001, **** *p* < 0.0001). Reproduced under the terms of the Creative Commons CC BY license from [107].

Fu et al. also reported a dual-drug PEGylated nanoliposome formulation (ARNIPL) that co-encapsulates ARV-825, a BRD4-targeting PROTAC, and nintedanib, a tumor stroma-modulating agent, aiming to enhance therapeutic efficacy against vemurafenib-resistant melanoma (Figure 7) [107]. By employing a modified hydration technique with citric acid, the researchers achieved a high encapsulation efficiency (over 90%) and drug loading, effectively tackling the poor aqueous solubility of both drugs (Figure 7a). The resultant nanosized platform (approximately 111 nm) demonstrated prolonged stability over a month and a sustained release profile without a burst phase, enabling controlled drug delivery. In vemurafenib-resistant melanoma cells (A375R), ARNIPL showed significantly improved cytotoxic effects and reduced IC_50_ values compared with free drugs, indicating strong synergy through BRD4 degradation, c-Myc suppression, and increased apoptosis. Three-dimensional tumor spheroid assays further demonstrated enhanced tissue penetration, decreased TGF-β1 expression, and notable tumor inhibition, underscoring nintedanib’s role in stromal remodeling. The study demonstrated the potential of liposomal formulation for the co-delivery of PROTAC and small molecules, enabling efficient and synergistic therapeutic effects.

Chen et al. developed a liposome-based co-delivery system for ARV-825 and docetaxel (DTX) to enhance breast cancer chemotherapy and immunotherapy [108]. The liposomal formulation increased drug solubility, raising ARV825 and DTX concentrations from <10 μg/mL to 1 mg/mL, while maintaining a particle size of ~115.7 nm and a zeta potential of −17.1 mV for stability. This co-delivery system enabled controlled drug release, with 98.9% encapsulation efficiency for DTX and 99.4% for ARV825, ensuring precise intracellular degradation of BRD4, downregulation of Bcl-2, and reduction in PD-L1 expression. In vivo studies show a tumor inhibition rate of 57.4% for the co-loaded liposomes, outperforming single-drug-loaded liposomal formulations, while reducing the systemic toxicity. Overall, the liposomal approach enhances drug exposure to disease targets, increases tumor accumulation by 2.5-fold via the EPR effect, and improves therapeutic efficacy, making it a promising strategy for combination cancer treatments.

The application of liposomal platforms for the delivery of PROTACs has shown potential for overcoming key challenges of PROTACs in cancer treatment, including poor solubility, limited drug exposure, and drug resistance. By encapsulating small-molecule inhibitors and PROTACs within tunable liposomes—often combined with targeting ligands or co-loaded with additional agents—researchers have achieved enhanced stability, prolonged circulation, and more efficient tumor accumulation via the EPR effect. However, despite these advantages, liposome-based delivery methods, like LNPs, face significant limitations, including a lack of selectivity, which can result in off-target effects, suboptimal therapeutic outcomes, and increased systemic toxicity. Additionally, liposomes are prone to enzymatic degradation, leading to premature drug leakage and reduced efficacy. This could potentially explain why the GALARV apoptotic effect is less than 50%. Moreover, DT@NPs, which have a prolonged drug release of up to 120 h, have shown potential for systemic toxicity.

To overcome these challenges, ligand-functionalized liposomes have been developed, like GALARV, conjugated with a ligand or DT2216 bound with siRNA, to facilitate receptor-mediated endocytosis and enhance cellular uptake. This method enables the efficient recognition and degradation of POI. Given these advantages, liposome-based delivery may offer a promising strategy for targeted drug delivery, selectively accumulating in designated cell types or organs that overexpress specific receptors.

### 3.6. Exosome-Based Delivery Systems

Exosomes are nano-sized extracellular vesicles that have gained attention as a possible source of biomarkers due to their capacity to transfer proteins, RNA, and DNA across cells [109]. Utilizing exosomes for PROTAC delivery represents a novel and promising technique that offers a potential remedy for the intricate problems associated with viral infections. Through the facilitation of targeted administration of PROTACs, this technique greatly reduces the off-target effects associated with standard therapies and ultimately improves the overall therapeutic outcomes. Exosomes offer distinct benefits such as inherent biocompatibility, minimal immunogenic responses, and the capacity to traverse biological barriers, including the blood-brain barrier, making them particularly promising carriers for drug delivery. Their cell-derived nature allows for the precise delivery of PROTACs to target cells, reducing systemic toxicity and enhancing therapeutic efficacy. This strategy efficiently manages issues, which are frequently seen with the use of standard PROTACs [110].

**Figure 8:**
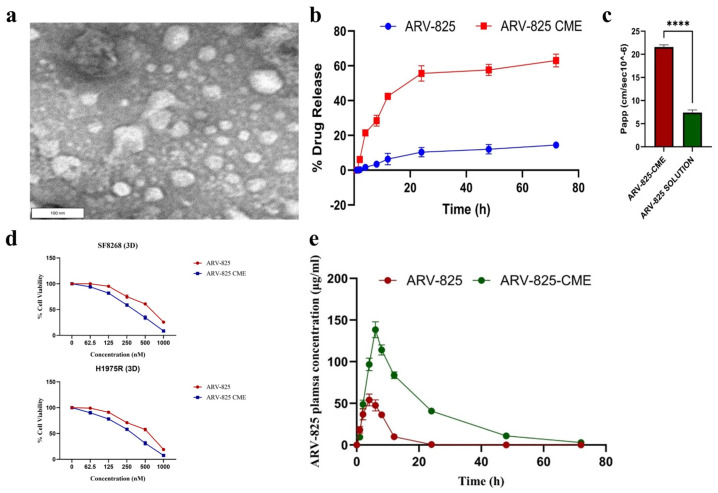
Characterization, drug release, permeability, cytotoxicity, and pharmacokinetics of camel milk-derived exosome (CME) containing ARV-825. (**a**) Morphology of ARV-825-CME (TEM image, 100 nm scale bar). (**b**) In vitro drug release in a pH 7.4 media. (**c**) Apparent permeability coefficient (Papp) of ARV-825-CME (**** *p* < 0.0001). (**d**) Concentration-dependent cell viability of SF8628 DIPG cells in 3D culture. (**e**) The plasma concentration of ARV-825 in rats after oral administration of ARV-825 and ARV-825-CME. Reproduced under the terms of the Creative Commons CC BY license from [111].

Nathani et al. investigated camel milk-derived exosomes (CMEs) as a novel drug delivery system for enhancing the bioavailability and therapeutic efficacy of ARV-825 PROTAC, a BRD4-targeting anticancer agent (Figure 8) [111]. The CME formulation exhibited an entrapment efficiency of 42.75% for ARV-825. The particle exhibited a size of 136.8 nm and a zeta potential measured at −32.75 mV, which provided stability and facilitated prolonged drug release. (Figure 8a,b). In vitro studies demonstrated a 5.4-fold increase in drug release kinetics and a 3.2-fold enhancement in permeability, leading to a reduction in IC_50_ values against resistant cancer cell lines (Figure 8c). In vivo pharmacokinetic studies revealed a two-fold increase in plasma concentration and a five-fold improvement in bioavailability, confirming better systemic absorption through the oral administration of the CME formulation (Figure 8d). Overall, CMEs offer a biocompatible and effective strategy for improving ARV-825 delivery, optimizing drug absorption, and enhancing anticancer efficacy with reduced off-target toxicity.

Exosomes have been proposed as a potential solution for delivering PROTACs to difficult-to-target cells. However, challenges remain in the development of exosome-based delivery systems for PROTACs. One of the potential obstacles is the difficulty in exosome production in sufficient quantities and with high purity. Additionally, the inherent loading capacity of exosomes for therapeutic cargo can be limited, as evidenced in a previous study showing that the entrapment efficiency of ARV-825 using CMEs is less than 50%. These limitations underscore the need for further optimization and validation of exosome-based delivery systems to fully realize their potential and application in PROTAC therapies.

## 4. Summary

PROTACs have generated considerable excitement in drug development by leveraging the cell’s endogenous ubiquitin–proteasome pathway to selectively degrade disease-related proteins. Despite their promise, PROTACs face multiple barriers to clinical translation, including a low aqueous solubility, high molecular weight, poor membrane permeability, and challenges in targeted cell delivery. These characteristics pose significant limitations for both parenteral and oral administrations, resulting in suboptimal absorption, rapid clearance, and reduced therapeutic efficacy.

To address these challenges, researchers have explored a variety of nanotechnology-based strategies aimed at improving the physicochemical and pharmacokinetic properties of PROTACs (Table 2). Polymeric nanoparticles have been widely studied due to their adaptable structure and the ability to encapsulate one or more hydrophobic agents in a single nanosized carrier. For instance, polymeric micelle systems improve the solubility and circulation time of poorly soluble PROTACs, enhance their tumor accumulation, and enable controlled release. Moreover, the chemical versatility of polymers facilitates additional modifications—such as ligand conjugation or stimuli-responsive elements—to achieve site-specific activation and enhanced cellular uptake. These polymeric approaches have demonstrated improved target protein degradation, although many reports emphasize the need for robust in vivo studies to confirm translational potential.

Emulsion-based systems, including SNEDDS, have also shown notable potential in enhancing the solubility and therapeutic performance of PROTACs. Recent studies indicate that SNEDDS-based strategies not only enhance the effects of PROTACs but also offer the potential for combination therapies, where multiple agents targeting complementary pathways are co-delivered within the same formulation.

ASDs represent another established formulation approach aimed at generating and maintaining drug supersaturation in the gastrointestinal tract. Through spray drying or hot melt extrusion, PROTACs can be incorporated into polymeric matrices, thereby increasing their apparent solubility and dissolution kinetics. While ASDs have successfully improved the absorption of numerous small-molecule drugs, applications in high-molecular-weight, beyond-Rule-of-Five PROTACs remain relatively nascent. Nonetheless, early evidence suggests that ASDs can retain PROTACs in a supersaturated state long enough to improve oral exposure, setting a foundation for future exploration of optimized excipient and process choices.

LNPs and liposomes offer complementary benefits, particularly with respect to biocompatibility, modifiable surface characteristics, and the capacity to encapsulate both hydrophilic and lipophilic agents. Multiple studies have demonstrated that lipid nanoparticles and liposomes can substantially raise the solubility of PROTACs, protect them from rapid clearance, and facilitate site-specific delivery via surface engineering. Liposomal co-delivery systems have further underscored the potential of combination therapies, notably in cancer treatment, where PROTACs are administered alongside chemotherapeutic or immunomodulatory agents. These strategies have led to synergistic antitumor effects, reduced off-target toxicity, and improved pharmacokinetic profiles, emphasizing the adaptability of lipid carriers for complex drug regimens.

Lastly, exosome-based delivery constitutes a cutting-edge approach with inherent advantages of low immunogenicity, natural biocompatibility, and the ability to cross challenging biological barriers. Emerging reports highlight the feasibility of loading PROTACs into exosomes derived from cell sources like camel milk. By capitalizing on exosomes’ endogenous targeting mechanisms, these formulations achieve improved drug release kinetics, enhanced intracellular uptake, and greater bioavailability, all of which are crucial for realizing the full therapeutic promise of PROTACs. Although still in its early stages, exosome-mediated delivery offers a unique platform that could be especially beneficial for complex or sensitive therapeutic cargo.

In summary, while the various nanoparticle and solid dispersion systems collectively aim to surmount the solubility and delivery challenges of PROTACs, each exhibits distinct strengths. Polymeric micelles, emulsions, and lipid-based carriers are proven to improve oral or systemic absorption, while ASDs provide a more straightforward, though less targeted, solution. Exosomes offer a novel biologically driven approach but still face manufacturing hurdles. By capitalizing on the similarities—such as achieving supersaturation or encapsulation of hydrophobic drugs—and harnessing the unique advantages of each platform, researchers can build robust, tailored delivery strategies that accelerate PROTACs toward clinical adoption.

## 5. Discussion and Future Perspective

PROTAC technology has significantly reshaped small-molecule drug development, offering a route to degrade disease-associated proteins previously deemed “undruggable”. However, their large molecular size, hydrophobic nature, and inherent complexity often hinder solubility, permeability, and pharmacokinetics—challenges that must be overcome to realize the full clinical potential of these novel therapeutics. Toward this goal, a range of nanotechnology-driven delivery strategies have emerged. Self-nanoemulsifying systems protect PROTACs from aqueous environments, polymeric nanoparticles and liposomes enable controlled release and longer circulation times, and stimuli-responsive formulations enhance site specificity while reducing off-target effects. Exosome-based carriers and multifunctional designs capable of receptor-mediated targeting or dual stimuli-responsiveness offer even greater promise for optimizing efficacy, toxicity, and biodistribution.

As PROTAC therapies move closer to wider clinical adoption, large-scale and cost-effective manufacturing of their delivery systems will be essential. Processes should be designed to ensure batch-to-batch consistency, reproducibility, and stringent quality control, which are non-negotiable requirements for regulatory approval. In parallel, attention must be paid to selecting excipients that are already recognized as safe by regulatory agencies such as the FDA. Employing established, well-characterized materials allows for a more straightforward regulatory pathway and accelerates the move from bench to bedside, ultimately expediting patient access to these novel treatments.

Clinical trial data have begun to underscore the importance of individualized dosing regimens and careful patient stratification. The pharmacokinetics of large, complex molecules like PROTACs can vary widely among different patient populations, influenced by factors such as metabolic rates, genetic polymorphisms, and disease states. Thus, future research must focus on personalized approaches, combining advanced drug delivery systems with predictive biomarkers to guide treatment decisions. This precision-medicine mindset will not only enhance therapeutic outcomes but also reduce side effects, ultimately improving patient quality of life.

An equally important focus is the development of an alternative delivery system for PROTACs, such as a mesoporous delivery system or carbon nanotube delivery system, which have gained attention in drug delivery research for delivering poorly soluble drugs like PROTACs [112,113]. Additionally, the formulation design of emerging heterobifunctional molecules similar to PROTACs, such as DUBTACs, AceTACs, and PhosTACs, is also a key area of interest [114,115,116]. While each of these platforms leverages distinct biological pathways for targeted protein modification or degradation, they share structural and physicochemical features that frequently fall outside Lipinski’s Rule of Five. Consequently, they face many of the same hurdles involving poor solubility, insufficient permeability, and inconsistent bioavailability. Leveraging advanced formulation technologies originally developed for PROTACs could streamline the development and clinical translation of these next-generation therapeutics, further extending the frontier of targeted protein degradation and modification.

## 6. Conclusions

In summary, the future success of PROTAC-based therapies hinges on a multidisciplinary effort that unites the fields of medicinal chemistry, nanotechnology, pharmaceutical sciences, and clinical investigation. By refining strategies to overcome solubility limitations, enhance tumor targeting, and mitigate off-target toxicity, these engineered protein degraders can achieve their full potential as a transformative force in treating both oncological and other complex diseases. As additional heterobifunctional molecules enter the pipeline, lessons learned from PROTAC development and formulation will guide their path, potentially expanding the scope of protein modulation therapies to new therapeutic areas. With concerted efforts in research, development, and clinical validation—paired with the use of safe, well-established excipients—PROTACs and similar modalities hold the promise of revolutionizing patient care in the decades to come.

## Figures and Tables

**Table 1:** PROTACs in clinical trial phases.

PROTAC	POI	E3 Ligase	Clinical Phase	ROA	Diseases	Target Type	Clinical Trial Number
ARV-110	AR	CRBN	Phase II/III	Oral	Prostate cancer	Nuclear receptor	NCT03888612
ARV-471	ER	CRBN	Phase III	Oral	Breast cancer	Nuclear receptor	NCT04072952
ARV-766	AR	-	Phase I/II	Oral	Prostate cancer	Nuclear receptor	NCT05067140
AC682	ER	CRBN	Phase I	Oral	Breast cancer	Nuclear receptor	NCT05080842
CC-94676	AR	CRBN	Phase I	Oral	Prostate cancer	Nuclear receptor	NCT04428788
DT2216	Bcl-xL	VHL	Phase I	I.V	Liquid and solid tumors	Anti-apoptotic protein	NCT04886622
FHD-609	BRD9	CRBN	Phase I	I.V	Synovial sarcoma	Nuclear protein	NCT04965753
KT-333	STAT3	-	Phase I	I.V	Liquid and solid tumors	Nuclear protein	NCT05225584
KT-413	IRAK4	CRBN	Phase I	I.V	DLBCL (MYD88-mutant)	Serine/threonine kinase	NCT05233033
KT-474	IRAK4	CRBN	Phase I	Oral	Autoimmune diseases	Serine/threonine kinase	NCT04772885
NX-2127	BTK	CRBN	Phase I	Oral	B cell malignancies	Non-receptor tyrosine kinase	NCT04830137
NX-5948	BTK	CRBN	Phase I	Oral	B cell malignancies and autoimmune diseases	Non-receptor tyrosine kinase	NCT05131022
CFT8634	BRD9	CRBN	Phase I/II	Oral	Synovial sarcoma	Nuclear protein	NCT05355573
CFT1946	BRAF-V600X	CRBN	Phase I/II	Oral	Solid tumors	Serine/threonine kinase	NCT05668585
CFT8919	EGFR-L858R	CRBN	Phase I	Oral	Non-small cell lung cancer (NSCLC)	Receptor tyrosine kinase	NCT06641609
CG001419	TRK	CRBN	Phase I	Oral	Cancer and other indications	Receptor tyrosine kinase	NCT06636500

**Table 2:** Delivery system for PROTAC and its improvement.

PROTAC	POI	E3 Ligase	Diseases/Cell Lines	Particle Size (nm)	Zeta Potential (mV)	Delivery System	Improvements	**Limitations**	**Ref.**
ARV-825	BRD4	-	Pancreatic cancer	89.63 ± 16.39	-	Polymeric nanoparticle	Prolonged half-life, enhanced cell permeability	-	[45]
ARV-825	BRD4	-	Glioma	26.3 ± 0.7	−13.3 ± 8.0	Polymeric nanoparticle	Penetrates BBB, increased stability, and reduced toxicity	Slow drug release (26.68% at 96 h)	[46]
MZ1	BRD4	-	HER2-positive breast cancer	114 ± 2.3	31.8 ± 0.5	Polymeric nanoparticle	Targeted delivery	-	[52]
dBET6	BRD4	CRBN	CD8+ T cells	-	-	Polymeric nanoparticle	Minimized toxicity and enhanced stability	No formulation stability data	[53]
ARV-825	BRD4	CRBN	Colorectal cancer	59.31	−0.64	Polymeric nanoparticle	Enhanced cell permeability and EPR effects	Drug release depends on redox-responsive release	[54]
ARV-771	BRD4	-	TNBC cells			Polymeric nanoparticle	Cell permeability enhancement	-	[57]
ARV-771	BRD4	VHL	HeLa and B16F10 cells	118	−32.1	Polymeric nanoparticle	Improved solubility and intracellular delivery	Unstable particle size, low reproducibility	[58]
dBET6	BRD4	CRBN	Lung cancer	229.71 ± 72.1	−29.0	Stimuli-responsive and NPs	Targeted delivery	Particle size is over 200 nm, high dependency on pH and GSH level	[59]
dBET6	BRD4	-	Lung cancer	-	-	Stimuli-responsive and NPs	High drug-loading capacity, improved stability	-	[60]
MS39	EGFR	VHL	HCC-827 and PC-9 cells	202 ± 1.7	−7.1 ± 0.12	Self-assembled NPs	Increased stability and cell permeability	Reproducibility issue	[61]
MZ1	BRD4	VHL	-	141.80 ± 5.66	-	Stimuli-responsive delivery	Enhanced permeability and EPR effect	Long and pricey development process	[62]
ARV-825	BRD4	CRBN	Vemurafenib-resistant melanoma cells	45.02	-3.78	SNEDDS	Enhanced solubility	Rapid precipitation, high concentration of DMA, stability relies on the selection and balance of excipients	[75]
ARV-825	BRD4	-	Vemurafenib-resistant melanoma cells	-	-	Emulsion	Enhanced solubility	-	[76]
ARCC-4	AR	VHL	-	-	-	ASDs	Enhanced solubility and stability	Low drug loading, high dependency on polymer and its concentration	[82]
ARV-110 and SelDeg51	-	-	-	-	-	ASDs	Enhanced solubility and stability	Long-term stability issues, low drug loading, pH-dependent dissolution profiles	[83]
MS4078	-	-	-	-	-	ASDs	Enhanced solubility and stability	Long-term stability issues	[84]
ARV-825	BRD4	CRBN	NSCLC	56.33 ± 0.42	−21 ± 1.24	LNPs	Improved solubility, stability, and intracellular delivery	Long-term stability issues	[93]
ARV-771	BRD4	VHL	HeLa cells	-	-	LNPs	Cell permeability enhancement	Low encapsulation efficiency	[94]
ARV-825	BRD4	-	BRAFi-resistant melanoma cells	100	-	LNPs	Lowered dosing and improved safety	-	[95]
DT2216	Bcl-xL	-	Cervical and breast cancer	~100	-	Liposome	Good bioavailability in cells, reduced off-target and side effect	systemic toxicity concerns due to long-term released (up to 120 h)	[103]
ARV-825	BRD4	CRBN	Vemurafenib-resistant melanoma cells	93.83 ± 10.05	−27.30	Liposome	Improved solubility and stability	Low apoptotic effect (<50%)	[104]
DT2216	Bcl-xL	-	-	200–300	-	Liposome	Improved solubility	Low encapsulation efficiency, formulation stability	[105]
ARV-825	BRD4	-	Vemurafenib-resistant melanoma cells	105.25 ± 2.76	26.6	Liposome	Enhanced stability and minimized side effects	-	[106]
ARV-825	BRD4	-	Vemurafenib-resistant melanoma cells	111.1 ± 6.55	13.9 ± 6.62	Liposome	Improved cell permeability and stability	Long-term stability issues	[107]
ARV-825	BRD4	-	Breast cancer	~115.7	−17.1	Liposome	Increased solubility, reduced systemic toxicity	-	[108]
ARV-825	-	-	-	136.8 ± 1.94	-	Exosome	Improved cellular uptake and cell permeability	Entrapment efficiency below 50%	[111]

## Abbreviations

ACNPs	Antibody-conjugated nanoparticles
AP-NLC	ARV-825-loaded PEGylated NLCs
API	Active pharmaceutical ingredient
AR	Androgen receptor
ARV-SNEP	ARV-825-loaded self-nanoemulsifying preconcentrate
ASDs	Amorphous solid dispersion
BBB	Blood–brain barrier
CME	Camel milk-derived exosome
CRBN	Cereblon
DS-PLGA	Disulfide-linked poly (lactic-co-glycolic acid)
EPR	Enhanced permeability and retention
ER	Estrogen receptor
GALARV	Galactose-decorated nanoliposomal formulation
GSH	Glutathione
HCC	Hepatocellular carcinoma
LLCM	Lung cancer cell membrane
LNP	Lipid nanoparticles
MPRO	Folate-PEG-PROTAC micelles
MSPM	Mixed-shell polymeric micelle
MZ1-NPs	MZ1-loaded polymeric nanoparticles
nChap	Nanochaperone-based
NLC	Nanostructured lipid carrier
NSCLC	Non-small cell lung cancer
PCL	Polycaprolactone
PDSA	Poly(disulfide amide)
PEG	Polyethylene glycol
PEI	Polyethyleneimine
PEO-PPO-PEO	Poly(ethylene oxide)-poly(propylene oxide)-poly(ethylene oxide)
PGDAT	Self-assembling PROTAC nanoparticle
PLA	Polylactide
PLGA	Poly(lactic-co-glycolic acid)
POI	Protein of interest
PROTAC	Proteolysis targeting chimeras
PVA	Polyvinyl alcohol
PVP	Polyvinylpyrrolidone
RCNprotac	X-ray radiation responsive PROTAC nanomicelle
ROS	Reactive oxygen species
SNEDDS	Self-nanoemulsifying drug delivery system
SP	Substance P
TAMs	Tumor-associated macrophages
TNBC	Triple-negative breast cancer
UPS	Ubiquitin–proteosome system
VHL	Von Hippel–Lindau
